# Knowledge and Awareness of Screening for Prostate Cancer Risk Factors and Symptoms Among the General Population in Tabuk City, Saudi Arabia

**DOI:** 10.7759/cureus.46472

**Published:** 2023-10-04

**Authors:** Tariq M Shaqran, Rawan M Alanazi, Alyaa M Haider, Amal D Almohammadi, Hassan A Hawsawi, Sultan G Almehmadi, Tareq B Alanaze, Meshari Y Al-Qahtani, Khalaf F Alshammari

**Affiliations:** 1 Family Medicine, King Salman Armed Forces Hospital, Tabuk, SAU; 2 College of Medicine, Dar Al Uloom University, Riyadh, SAU; 3 Faculty of Medicine, King Abdulaziz University, Jeddah, SAU; 4 Medical School, Taibah University, Al-Madinah, SAU; 5 Rabigh Faculty of Medicine, King Abdulaziz University, Jeddah, SAU; 6 Urology, Faculty of Medicine King Abdulaziz University, Jeddah, SAU; 7 General Medicine, University of Tabuk, Tabuk, SAU; 8 College of Medicine, King Khalid University, Abha, SAU; 9 Internal Medicine, King Salman Specialist Hospital, Hail, SAU

**Keywords:** tabuk, men, psa, knowledge survey, cancer, prostate

## Abstract

Background

Early-stage prostate cancer may not show any signs. Digital rectal examination and the prostate-specific antigen test are frequently used in the screening for prostate cancer. The objective of this research is to assess the knowledge and awareness of screening prostate cancer among males in Tabuk, Saudi Arabia.

Methodology

A cross-sectional study was performed among Saudi males in Tabuk City. A structured interviewing technique based on a questionnaire was used based on the objectives and research questions. Data were collected by well-trained data collectors from the general population in Tabuk City who were randomly chosen in proportion to the city’s population density. A multivariate logistic regression analysis was done to evaluate the variables related to knowledge and awareness in this study.

Results

This questionnaire was completed by a total of 417 male participants. In the studied group, 86.8% of participants had heard about prostate cancer through friends (59%), TV/radio/newspaper (53.24%), and other health services (41.49%). In addition, around 67.6% of participants knew about the prostate cancer screening test. In addition, 32.4% of participants had no prior knowledge of prostate cancer or a screening test.

Conclusions

There was a good level of awareness and attitude toward screening methods for prostate cancer (54.7%). Aside from having good knowledge regarding prostate cancer symptoms among males in Tabuk City, all participants with regard to demographic distribution showed a significant level of good knowledge and awareness of screening prostate cancer methods and the necessity of performing regular prostate examinations.

## Introduction

The male genitalia includes a tiny accessory gland located close to the lower part of the bladder known as the prostate gland, which undergoes malignant changes in prostate cancer. Proteolytic enzymes are released by the prostate, which weighs around 20 g, into the sperm to aid in fertilization [1]. Prostate cancer presents with physical and psychological symptoms that distinguish it. Early on, it is typically asymptomatic, but later on, symptoms including frequency, interrupted flow, nocturia, hematuria, and dysuria appear that are comparable to those of benign prostatic hyperplasia. Hip, spine, or rib discomfort can result from prostate cancer with bone metastasis [2,3].

The World Health Organization reports that prostate cancer incidence and fatality rates, notably in the Arab region, have increased and will likely continue to do so [4]. However, it was found that in advanced stages, progression was more common in the Arab region than in the United States [5]. The prostate cancer fatality rate in the United States has reduced by 40% since 1990, which is attributable to better screening procedures and treatment [6]. Prostate cancer is currently known as the second most common carcinoma in Saudi Arabia among males over the age of 60 years. Between 2001 and 2008 in Saudi Arabia, an age-standardized incidence rate of prostate cancer was anticipated to be 5.1 cases per 100,000 males [7].

Arabic, Eastern, and Asian ethnic groups have different prevalence rates for prostate cancer. The United States and Canada have the greatest incidence rates, followed by Europe. While the incidence rate is lower in Asian countries, especially among Arabic communities, the incidence rate according to age standardization of prostate cancer in the Kingdom of Saudi Arabia was 4.5/100,000 in 2012, with the Riyadh and Eastern regions seeing the highest rates. Compared to Europe and the Gulf, this incidence rate is significantly lower [8].

Although there is no single cause of prostate cancer, several factors can have an impact. These components include age, with men over the age of 50 accounting for 60% of new cases of prostate cancer, while cases in people under the age of 40 are rare. The chance of developing prostate cancer doubles when a family member already has the disease because it can run in families [9].

Race is also an essential issue as the illness is less frequent in Asia and more common in European nations (particularly northern regions of Europe) and North America due to a variety of variables such as lifestyle and food. Indeed, genetic variations can play a significant role, as mutations in a cell’s DNA cause the cell to become malignant [9,10].

Of note, the relationship between perfect awareness of prostate carcinoma screening and contradictions is unclear. Male people who decided not to be tested had less significant awareness of prostate carcinoma and a less favorable attitude toward different screening methods [11]. This information suggests that educating men about screening might help increase screening rates. Other studies found that once participants were aware of the benefits and expenses of different screening methods, educational interventions decreased their interest in the procedure [12].

Regular checkups for prostate cancer can result in an early diagnosis, reducing the likelihood of negative outcomes; nevertheless, screening practices differ by population. An American study found that hurdles to prostate cancer screening include socioeconomic status, fear, communication between patients and doctors, skepticism of the medical community, and resistance to digital rectal examinations [13].

It is unclear why males refuse to undergo prostate cancer screening. Studies discussing this issue among public people in the Arabic region are rare. The incidence of prostate cancer in developing countries varies significantly from that in the United States and Europe. Additionally, there is no national screening scheme in Arabic countries. More information on the factors that affect attendees is required to alter screening processes. This research analyzed men’s awareness and attitudes about prostate cancer and its screening modalities in our region to identify the potential factors that contribute to better prostate cancer screening [14].

## Materials and methods

Study design

This study was conducted in Tabuk City, Saudi Arabia. The general population’s knowledge and awareness of screening for prostate cancer risk factors and symptoms were examined using a cross-sectional survey methodology between May and August 2023.

Study population

This study included all adults 40 years of age and older living in Tabuk City who were willing to participate. Individuals aged less than 40 years, those who had genitalia disease, and all women were excluded.

Sample size

The sample size for this study was determined using the following formula: n = (Z^2^ × p × q)/d^2^, where n is the required sample size, Z is the confidence level (standard value of 1.96 for 95% confidence), p is the estimated percentage of the population, q is 1-p, and d is the margin of error (5% or 0.05). We used a prevalence rate of 50% based on previous studies. The minimum sample size required for this study was calculated at 384 participants. We recruited 417 participants in this study.

Sampling technique

This study employed random sampling as its sampling strategy. Participants were chosen at random from the general population of Tabuk City’s general center. The city was split into neighborhoods, and participants were chosen from each of them. This approach guaranteed that the sample was representative of the population and that there were enough participants.

Data collection tools

The data were gathered face-to-face using a questionnaire based on the objectives and study questions (see Appendices). The questionnaire was developed in English, and a committee of specialists reviewed it and made changes. To prevent variance in data collection, data collectors were trained on how to Arabize the questions in a session designed specifically for this purpose. To verify its clarity and validity, a pilot study was conducted among 23 people from the target group who were not included in the final analysis. Cronbach’s alpha was 0.74, which was acceptable.

Data analysis

SPSS version 28 (IBM Corp., Armonk, NY, USA) was used to analyze the data. Data were compiled using descriptive statistics. The association between knowledge and awareness of screening for prostate cancer risk factors, symptoms, and demographic characteristics was examined using the chi-square test and logistic regression analysis. A p-value ≤0.05 was considered significant.

Ethical considerations

The Institutional Review Board of King Salman Armed Forces Hospital provided ethical permission for this study (approval number: KSAFH-REC-2023-515). All study participants provided verbal informed consent before participating in the study, and the questionnaire was gathered from respondents and analyzed with secrecy while ensuring privacy.

## Results

A total of 417 male participants were included in this study, with ages ranging from 40 to over 60 years old. The sociodemographic characteristics of participants are illustrated in Table 1.

**Table 1:** Sociodemographic characteristics of the study participants (n = 417).

Variable	Response	N	%
Age groups (years)	40–49	245	58.8
	50–59	90	21.6
	60 years and above	82	19.6
Marital status	Single	95	22.8
	Married	242	58.0
	Divorced	52	12.5
	Widowed	28	6.7
Education level	Elementary school	23	5.5
	Middle school	48	11.5
	High school	86	20.6
	University	194	46.5
	Higher education	49	11.8
	No education	17	4.1
Occupation	Employed	255	61.2
	Unemployed	66	15.8
	Retired	96	23.0
Monthly income	Less than 5,000 SAR	81	19.4
	5,000–9,999 SAR	152	36.5
	10,000–14,999 SAR	89	21.3
	Above 15,000 SAR	95	22.8

Table 2 presents the participants’ knowledge of prostate cancer symptoms and risk factors. Participants had a good understanding of prostate cancer (n = 362, 86.6%). Friends were the most common source of information (n = 246, 58.99%), followed by other health services (n = 173, 41.49%). The majority of participants demonstrated inadequate knowledge about how to prevent prostate cancer (n = 256, 61.4%), and family history of the disease was the risk factor with the highest frequency (n = 300, 71.94%), followed by age (n = 273, 65.47%) between 40 and 50 years. Age over 50 years old was the group with the highest frequency as the age when they should be more concerned about getting an examination (n = 172, 41.2%).

**Table 2:** Knowledge of prostate cancer risk factors and symptoms.

Question	Response	N	%
Have you heard about prostate cancer?	Yes	362	86.8
	No	55	13.2
If yes, where/who mentioned it?	Friends	246	58.99
	Other health service	173	41.49
	Relatives	137	32.85
	PSF (family health program)	158	37.89
	Other	26	6.24
	TV/Radio/Newspaper	222	53.24
What are the risk factors for prostate cancer?	Age	273	65.47
	A family history of prostate cancer	300	71.94
	Obesity	149	35.73
	Smoking	218	52.28
	Race/ethnicity	159	38.13
	None of the above	20	4.80
Can prostate cancer be prevented?	Yes	130	31.2
	No	31	7.4
	Not sure	256	61.4
What are the symptoms of prostate cancer?	Blood in the urine or semen	285	68.35
	Pain or stiffness in the lower back, hips, or thighs	134	32.13
	Painful ejaculation	176	42.21
	Difficulty urinating	311	74.58
	Weak or interrupted urine flow	267	64.03
	None of the above	26	6.24
In your opinion, at what age should men be more concerned to undergo the examination?	>50 years old	172	41.2
	30–<40 years old	71	17.0
	40–50 years old	122	29.3
	Do not know	52	12.5

As shown in Table 3, 417 men completed the questionnaire. The majority had a high awareness of prostate cancer (n = 362, 86.8%) (p = 0.001): How crucial is it to undergo routine prostate exams? (p ≤ 0.0001); has a doctor ever suggested that you get screened for prostate cancer? (p = 0.01); and when was the last time you underwent PSA screening? (p = 0.002).

**Table 3:** Awareness of participants for prostate cancer screening. ***: p < 0.001 is statistically significant. The chi-square test was done. **: p < 0.01 is statistically significant. The chi-square test was done. *: p < 0.05 is statistically significant. The chi-square test was done. PSA: prostate-specific antigen

Question	Response	Have you heard about prostate cancer?			
		N 417	Yes 362 (86.8%)	No 55 (13.2%)	P-value
Do you know some kinds of examinations for cancer detection?	Yes	282	264 (72.9%)	18 (32.7%)	0.001**
	No	135	98 (27.1%)	37 (67.3%)	
If yes, what types of examinations do you know?	PSA blood test	59	54 (14.9%)	5 (9.1%)	0.16
	Rectal exam	125	110 (30.4%)	15 (27.3%)	
	Rectal exam/PSA blood test	233	198 (54.7%)	35 (63.6%)	
Is prostate examination the only way to diagnose prostate cancer?	Yes	106	94 (26%)	12 (21.8%)	0.164
	No	99	90 (24.9%)	9 (16.4%)	
	Do not know	212	178 (49.2%)	34 (61.8%)	
Is the adequate frequency of screening for men the same age as the interviewers annually?	Every three to five years	123	108 (29.8%)	15 (27.3%)	0.624
	Every two years	102	88 (24.3%)	14 (25.5%)	
	Only when there are symptoms	73	66 (18.2%)	7 (12.7%)	
	Do not know	119	100 (27.6%)	19 (34.5%)	
Should only men with urinary symptoms undergo screening?	Yes	258	230 (63.5%)	28(50.9%)	0.122
	No	42	33 (9.1%)	9 (16.4%)	
	Do not know	117	99 (27.3%)	18 (32.7%)	
How important is it to perform prostate examinations regularly?	Doesn’t matter	37	24 (6.6%)	13 (23.6%)	<0.0001***
	Important	320	291 (80.4%)	29 (52.7%)	
	Little or not important at all	60	47 (13%)	13 (23.6%)	
Has any physician advised you to undergo screening for prostate cancer?	Yes	141	132 (36.5%)	9 (16.4%)	0.01**
	No	167	137 (37.8%)	30 (54.5%)	
	Do not know/Do not remember	109	93 (25.7%)	16 (29.1%)	
Have you ever undergone a prostate examination?	Yes	102	95 (26.2%)	7 (12.7%)	0.029*
	No	229	190 (52.5%)	39 (70.9%)	
	Do not know/Do not remember	86	77 (21.3%)	9 (16.4%)	
What is the reason for the request for a prostate examination?	Cancer case in the family	46	38 (10.5%)	8 (14.5%)	0.446
	Presented symptoms	137	117 (32.3%)	20 (36.4%)	
	Prevention	153	138 (38.1%)	15 (27.3%)	
	The participant requested the examination	81	69 (19.1%)	12 (21.8%)	
When was the last time you underwent the examination?	Between one and two years	62	53 (14.6%)	9 (16.4%)	0.65
	Less than one year ago	25	20 (5.5%)	5 (9.1%)	
	Never	253	220 (60.8%)	33 (60%)	
	Over three years ago	77	69 (19.1%)	8 (14.5%)	
Have you ever undergone a PSA?	Yes	57	48 (13.3%)	9 (16.4%)	0.248
	No	237	202 (55.9%)	35 (63.6%)	
	Do not know/Do not remember	123	112 (30.9%)	11 (20%)	
When was the last time you underwent a PSA?	Between one and two years	24	20 (5.5%)	4 (7.3%)	0.002**
	Less than one year ago	20	13 (3.6%)	7 (12.7%)	
	Never	330	296 (81.8%)	34 (61.8%)	
	Over three years ago	43	33 (9.1%)	10 (18.2%)	

When participants were asked whether they had heard of prostate cancer, there was a non-significant correlation between their responses and the following questions: If yes, what kinds of examinations are you familiar with? (p = 0.160); can prostate cancer only be detected through a prostate exam? (p = 0.164); is the annual screening frequency for men the same as the interviewees’ average age? (p = 0.624); should only men with urological symptoms be screened? (p=0.122), what prompted the request for the prostate exam? (p = 0.446); when did you most recently have the test? (p=0.650); have you ever had a PSA performed? (p = 0.248).

## Discussion

The cancer that most commonly affects adult men worldwide is prostate cancer, which can be fatal if there is any delay in diagnosis. It is thought to be the second most common type of cancer detected and is a factor in the rising death rate among adult males. Due to their limited awareness of the symptoms and their attitudes toward early screening, studies have shown that older men have a high prevalence of prostate cancer [15]. In Tabuk City, Saudi Arabia, this study examined the awareness, attitudes, and prostate cancer screening practices of men.

Studies conducted in Medina, Jeddah, and Makkah in 2022 [7] reported that the overall knowledge of prostate cancer was low at 47.5% and the awareness of prostate cancer screening tests was not known in 80.9%. A study conducted in Riyadh in 2015 [14] found that participants had insufficient awareness of prostate cancer risk factors, and reported that participants were characterized by having poor knowledge of prostate cancer detection where the mean of total correct knowledge was 51.2%. The vast majority of participants in our study (86.2%) had heard of and reported adequate knowledge about prostate cancer risk factors. Additionally, the majority of participants demonstrated an acceptable understanding of the signs of prostate cancer. Most study participants demonstrated good knowledge about prostate cancer screening.

Our results were in line with a study conducted in Oman (2020) [16]. The majority of participants reported that they obtained most of their knowledge about prostate cancer through friends (n = 246, 58,99%), followed by TV/radio/and newspapers (n = 222, 53.24%). Participants in this study were more aware of the signs and symptoms of prostate cancer than the general community in Tabuk, which indicated high levels of knowledge, in comparison to populations in other regions of Saudi Arabia and other nations like Jordan and Egypt [17].

Our study group revealed that smoking could be a significant risk factor for prostate cancer (n = 218, 52.3%), which was in contrast to previous studies by Quaife et al. [18] and Al-Fayez et al. [19] on the causal relationship between tobacco and prostate cancer. Our participants’ awareness of the risk factors for prostate cancer was greater (n = 300, 71.94%) for family history of the disease and advanced age (n = 273, 65.47%), and earlier investigations confirmed our findings such as the study by Quaife et al. [18].

Regarding demographic parameters, all participants in this study demonstrated appropriate awareness of prostate cancer. Our findings showed that the participants’ responses regarding the significance of performing routine prostate exams were as follows: all age groups agreed that performing routine prostate exams is important; regardless of marital status, all groups indicated that performing routine prostate exams is crucial.

All educational levels in the groups demonstrated the significance of undergoing prostate examinations regularly, except those with elementary and no education levels, who responded insufficiently. Regular prostate examinations should be performed, according to data from all occupational levels. A previous study found that participants in all monthly income categories significantly agreed that it is important to undergo prostate examinations on a regular basis, as supported by our findings.

Limitations of the study

The study sample might not be entirely representative of the study community because it was chosen at random, and the findings might not apply to other communities outside of Tabuk City.

## Conclusions

The majority of people in Tabuk City demonstrated commendable knowledge and a favorable attitude toward prostate cancer. However, a proportion of the population had a poor grasp, a pessimistic outlook, and an unfavorable opinion of prostate cancer screening and treatments. This emphasizes the requirement for more comprehensive educational activities that support screening behavior and early presentation. According to this study, a higher level of education is associated with a better comprehension of the causes and signs of prostate cancer.

## Appendices

**Table 4:** The questionnaire used in this study.

Section 1: Demographic information		
Variable	Response	
Age groups (years)	40–49	
	50–59	
	60 years and above	
Marital status	Single	
	Married	
	Divorced	
	Widowed	
Education level	Elementary school	
	Middle school	
	High school	
	University	
	Higher education	
	No education	
Occupation	Employed	
	Unemployed	
	Retired	
Monthly income	Less than 5,000 SAR	
	5000–9,999 SAR	
	10,000–14,999 SAR	
	Above 15,000 SAR	
Section 2: Knowledge of participants about prostate cancer symptoms and risk factors		
Question	Response	
Have you heard about prostate cancer?	Yes	
	No	
If yes, where/who mentioned it?	Friends	
	Other health service	
	Relatives	
	PSF (family health program)	
	Other	
	TV/Radio/Newspaper	
What are the risk factors for prostate cancer?	Age	
	Family history of prostate cancer	
	Obesity	
	Smoking	
	Race/ethnicity	
	None of the above	
Can prostate cancer be prevented?	Yes	
	No	
	Not sure	
What are the symptoms of prostate cancer?	Blood in the urine or semen	
	Pain or stiffness in the lower back, hips, or thighs	
	Painful ejaculation	
	Difficulty urinating	
	Weak or interrupted urine flow	
	None of the above	
In your opinion, at what age should men be more concerned to undergo the examination?	>50 years old	
	30–<40 years old	
	40–50 years old	
	Does not know	
Section 3: Awareness of participants for prostate cancer screening		
Question	Response	
		Do you know some kinds of examinations for cancer detection?	Yes	
No		
If yes, what types of examinations do you know?	PSA blood test	
	Rectal exam	
	Rectal exam/PSA blood test	
Is prostate examination the only way to diagnose prostate cancer?	Yes	
	No	
	Does not know	
Is the adequate frequency of screening for men the same age as the interviewers annually?	Every three to five years	
	Every two years	
	Only when there are symptoms	
	Do not know	
Should only those men with urinary symptoms undergo screening?	Yes	
	No	
	Do not know	
How important is it to perform prostate examinations regularly?	Doesn’t matter	
	Important	
	Little or not important at all	
Has any physician advised you to screen for prostate cancer?	Yes	
	No	
	Do not know/Do not remember	
Have you ever performed a prostate examination?	Yes	
	No	
	Does not know/Does not remember	
What is the reason for the request for a prostate examination?	Cancer case in the family	
	Presented symptoms	
	Prevention	
	The participant requested the examination	
When was the last time you underwent the examination?	Between one and two years	
	Less than one year ago	
	Never	
	Over three years ago	
Have you ever undergone a PSA?	Yes	
	No	
	Do not know/Do not remember	
When was the last time you underwent a PSA test?	Between one and two years	
	Less than one year ago	
	Never	
	Over three years ago	
